# Transplantation of Specific Human Astrocytes Promotes Functional Recovery after Spinal Cord Injury

**DOI:** 10.1371/journal.pone.0017328

**Published:** 2011-03-02

**Authors:** Stephen J. A. Davies, Chung-Hsuan Shih, Mark Noble, Margot Mayer-Proschel, Jeannette E. Davies, Christoph Proschel

**Affiliations:** 1 Department of Neurosurgery, University of Colorado Denver, Anschutz Medical Campus, Aurora, Colorado, United States of America; 2 Department of Biomedical Genetics, Institute for Stem Cell and Regenerative Medicine, University of Rochester Medical Center, Rochester, New York, United States of America; University of North Dakota, United States of America

## Abstract

Repairing trauma to the central nervous system by replacement of glial support cells is an increasingly attractive therapeutic strategy. We have focused on the less-studied replacement of astrocytes, the major support cell in the central nervous system, by generating astrocytes from embryonic human glial precursor cells using two different astrocyte differentiation inducing factors. The resulting astrocytes differed in expression of multiple proteins thought to either promote or inhibit central nervous system homeostasis and regeneration. When transplanted into acute transection injuries of the adult rat spinal cord, astrocytes generated by exposing human glial precursor cells to bone morphogenetic protein promoted significant recovery of volitional foot placement, axonal growth and notably robust increases in neuronal survival in multiple spinal cord laminae. In marked contrast, human glial precursor cells and astrocytes generated from these cells by exposure to ciliary neurotrophic factor both failed to promote significant behavioral recovery or similarly robust neuronal survival and support of axon growth at sites of injury. Our studies thus demonstrate functional differences between human astrocyte populations and suggest that pre-differentiation of precursor cells into a specific astrocyte subtype is required to optimize astrocyte replacement therapies. To our knowledge, this study is the first to show functional differences in ability to promote repair of the injured adult central nervous system between two distinct subtypes of human astrocytes derived from a common fetal glial precursor population. These findings are consistent with our previous studies of transplanting specific subtypes of rodent glial precursor derived astrocytes into sites of spinal cord injury, and indicate a remarkable conservation from rat to human of functional differences between astrocyte subtypes. In addition, our studies provide a specific population of human astrocytes that appears to be particularly suitable for further development towards clinical application in treating the traumatically injured or diseased human central nervous system.

## Introduction

The recognition that astrocyte dysfunction may play an important role in a wide range of neurological disorders raises the question of whether astrocyte transplantation could be of therapeutic value in treating the injured or diseased human central nervous system (CNS). For example, it has long been known that astrocytes within glial scar tissue contribute to the failure of axon regeneration across sites of traumatic brain or spinal cord injury [1]–[6]. A failure of normal astrocyte generation by CNS precursor cells has been discovered to be a consequence of the mutations that cause Vanishing White Matter leukodystrophy [7], and dysfunction of astrocytes has also been suggested to be of importance in models of amyotrophic lateral sclerosis [8], forebrain ischemic injury [9], epileptic seizures [10], Huntington's disease [11], tuberous sclerosis [12] and Rett syndrome [13]. We therefore have proposed that enhancing astrocyte function through transplantation of specific subtypes of astrocytes derived from glial precursors will promote repair and functional recovery after CNS injury [14].

There are a number of challenges inherent in the development of astrocyte-based treatments for human disease. One of the most important of these is the question of whether all astrocytes are equivalent in their ability to promote repair, or whether specific populations of astrocytes are more useful than others. While previous studies had demonstrated a synergistic effect of BMP and LIF on the astrocytic differentiation of human neural stem cells [15], it remains unclear whether BMP and LIF induce distinct types of astrocytes and if so, what the functional properties of these astrocytes may be with respect to repairing CNS injuries. The recent description of considerable astrocyte heterogeneity in the human CNS raises the question whether distinct astrocytes can also be derived from single populations of human glial precursors, and more importantly whether different human astrocyte populations exhibit distinct functional properties [16]. Further challenges include the identification of signaling molecules that promote the generation of beneficial populations of astrocytes, identification of appropriate stem and/or progenitor cell populations that can be the source of such cells, and determination of whether there are situations in which it is more useful to transplant astrocytes themselves rather than to transplant stem or progenitor cells that might generate astrocytes in vivo in response to signals present in the host environment.

We now show that astrocytes generated from the same population of human fetal glial precursor cells, by exposure to either bone morphogenetic protein (BMP) or ciliary neurotrophic factor (CNTF), promote widely divergent outcomes with respect to repairing the injured adult spinal cord. Transplantation of astrocytes generated by exposure of human glial progenitor cells (hGPCs) to BMP (hGDAs^BMP^) promoted robust behavioral recovery and multi-laminae protection of spinal cord neurons following spinal cord injury (SCI), while transplantation of undifferentiated hGPCs or astrocytes generated by hGPC exposure to CNTF (hGDAs^CNTF^) failed to provide such benefits. These results provide a defined population of human astrocytes suitable for further pre-clinical development for treatment of SCI, and demonstrate that pre-differentiation into astrocytes prior to transplantation provides a much greater functional recovery than transplantation of precursor cells themselves. Our results also underscore the importance of function-based analysis of astrocyte diversity as a foundation for the development of astrocyte transplantation-based therapies.

## Results

### Human glial precursors give rise to two distinct astrocyte populations in vitro

**Figure 1:**
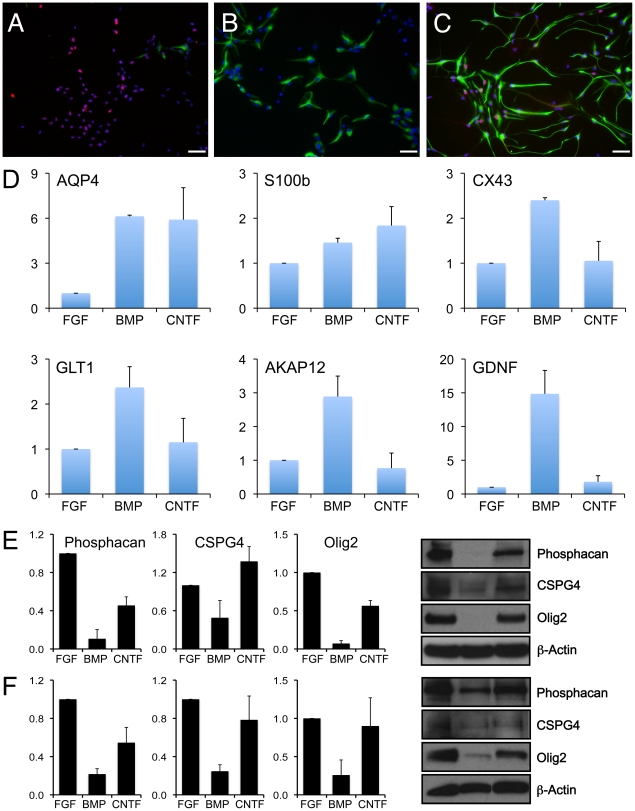
Generation of two distinct types of astrocytes from human glial precursors after treatment with BMP4 or CNTF. Both BMP and CNTF promote the differentiation of hGPCs into GFAP-positive astrocytes expressing S100b but with otherwise distinct morphological and antigenic phenotypes. hGDAs^BMP^ express lower levels of GFAP and exhibit a more compact morphology. hGDAs^CNTF^ have a more elongated morphology and expressed high levels of GFAP. hGDAs^CNTF^ also expressed high levels of neurite-outgrowth inhibitory chondroitin sulfate proteoglycans, phosphacan and CSPG4, as well as the transcription factor Olig2 - all of which have been found to be upregulated in glial scar associated astrocytes. (A–C) Human GPCs grown in bFGF (A) were induced to differentiate into astrocytes using BMP-4 (B) or CNTF (C). Labeling with anti-GFAP (Alexa-488) demonstrates that both BMP4 and CNTF induce differentiation of human glial precursors into GFAP-expressing astrocytes, while Olig2 expression (Alexa-568) is repressed in hGDAs^BMP^. Scale bar = 50 µm. (D) RT-QPCR analysis of hGPC, hGDA^BMP^ and hGDA^CNTF^ populations reveals induction of AQP4 and S100β in both hGDAs^BMP^ and hGDAs^CNTF^. Induction of CX43, GLT1, AKAP12 and GDNF however are restricted to hGDAs^BMP^. Average fold change and SD of expression levels is shown for three independent experiments using 9W-1 hGPCs. (E and F) Phosphacan and CSPG4 remain elevated in hGDAs^CNTF^ and are reduced in hGDAs^BMP^ derived from both 9W-1 (E) and 9W-2 (F) glial precursors. Mean relative protein expression and SD from three independent experiments are shown. Values were normalized to β-actin and expression in hGPCs.

As a first step towards determining whether human glial progenitor cells (hGPCs) can generate functionally distinct astrocyte populations, we exposed embryonic hGPCs isolated from spinal cords of 9.5 week old abortuses to BMP or CNTF. Both BMP and CNTF-induced astrocyte populations express GFAP (Fig. 1 A–C), AQP4 and S100β (Fig. 1 D), three widely used markers of astrocyte differentiation [17]–[22]. hGDAs^BMP^ also expressed connexin 43 (CX43), glutamate transporter 1 (GLT-1), AKAP12 and glia-derived neurotrophic factor (GDNF), all of which are expressed in the astroglial lineage [23]–[25]. In contrast, CNTF treatment did not induce expression of GLT-1, connexin 43, AKAP12, or GDNF. Instead, hGDAs^CNTF^ – but not hGDAs^BMP^ – expressed several antigens expressed in astrocytes generated in response to injury (Fig. 1 E and F), including the transcription factor OLIG2 and the chondroitin sulphate proteogylcans (CSPGs) phosphacan and CSPG4/NG2 [5], [26]–[31]. Thus, induction of differentiation of human spinal cord derived glial precursors with BMP or CNTF induces the generation of two phenotypically distinct astrocyte populations.

### Transplant morphology

To test the functional properties of these distinct astrocyte populations in vivo, hGDAs^BMP^, hGDAs^CNTF^ or undifferentiated hGPCs were transplanted into the injury site of adult Sprague-Dawley rats that had received unilateral transections of the right-side dorso-lateral funiculus (DLF), including the rubrospinal pathway, at the C3/C4 intervertebral spinal cord level.

**Figure 2:**
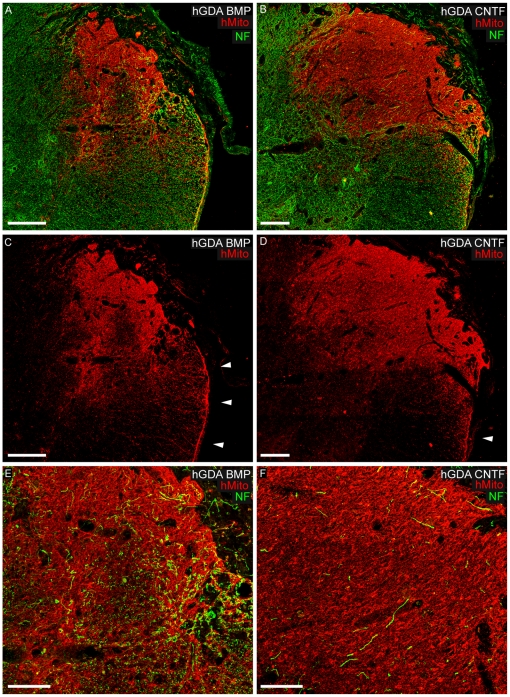
Human GDA graft survival and support of axon growth within spinal cord DLF injuries. Immuno-staining for human mitochondrial marker (red channel) of histological cross sections at sites of injury revealed hGDAs^BMP^ (A, C, E) and hGDAs^CNTF^ (B, D, F) transplant masses spanning the dorsal-ventral and lateral-medial margins of injury sites. Arrowheads in C and D indicate accumulations of hGDAs^BMP^ and hGDAs^CNTF^ respectively at the pial surface of lateral funiculus white matter (see also Fig. 4 A, B). Co-labeling for neurofilament (NF: green) and human mitochondrial marker (hMito: red) shows a markedly higher density of axons within hGDAs^BMP^ treated injury sites (A, E) compared to hGDAs^CNTF^ treated injury sites (B, F). Greater numbers of NF+ axons within hGDAs^BMP^ transplants aligned with the normal rostral/caudal trajectory of DLF white matter (E) compared to NF+ axons within hGDAs^CNTF^ transplants (F). Survival = 5 weeks post injury/transplantation. Scale bars: A, B, C, D = 200 µm; E, F = 100 µm.

Serial section analysis of transplants using antibodies to human mitochondria (hMito) showed that the majority of hGDA^BMP^ transplants (4 out of 6) and half of the hGDA^CNTF^ transplants (3 out of 6) that under went histological analysis had robust survival of hMito+ cells at 5 weeks post transplantation within dorsolateral funiculus (DLF) injury sites. Surviving transplants spanned the rostral to caudal extent of injury sites to effectively provide continuous substrates for potential growth of host axons across sites of injury. Qualitative assessment of transplant size showed that all surviving hGDA^CNTF^ transplants were larger than hGDA^BMP^ transplants in terms of both their rostral to caudal and lateral to medial extents (Fig. 2 A–D).

We next found that hGDA^BMP^ grafted injury sites exhibited higher densities of axons than hGDA^CNTF^ grafted injury sites. As shown in figure 2, hGDA^BMP^ grafts contained many 200 kD neurofilament+ axons (Fig. 2 A and E), while relatively few axons were observed in hGDA^CNTF^ grafts (Fig. 2 B and F). Quantification of neurofilament immuno-reactivity within hGDAs^BMP^ and hGDAs^CNTF^ treated injury sites revealed that the injury centers of hGDA^BMP^ treated spinal cords contained almost double the density of NF+ axon profiles (1.91 fold more: average 14.79 units^2^ +/− 3.05 st. dev.) than measured in hGDA^CNTF^ treated injury centers (average 7.75 units^2^ +/− 2.28 st. dev.; p = 0.003). Notably the profiles of the majority of NF+ axons, particularly within the rostral and caudal margins of hGDA^BMP^ grafted injuries, were end on to the transverse plane of section indicating that, as previously observed for axons within spinal cord injury sites bridged with rodent GDAs^BMP^ [14], these axons were aligned with the normal rostral/caudal trajectory of the DLF white matter (Fig. 2 A and E).

### hGDA migration and cell morphology

Analysis of the migration of transplanted hMito+ hGDAs revealed similar patterns of distribution within spinal cord gray matter. The highest densities of both types of hGDAs were observed within laminae 4, 5 and 6 of gray matter directly adjacent to sites of injury (Fig. 2 A–D). In contrast, relatively few hMito+ hGDA cells of either type were observed within laminae 7, 8 and 9 (Fig. 2 A–D) directly adjacent to injury sites. No hMito+ cells of either type of hGDA were observed to have migrated medially in either gray or white matter beyond the central canal i.e. into the contra-lateral side of the spinal cord. Significantly, in all hGDA treated cords analyzed, neither type of hGDA was found in the contra-lateral side of the spinal cord or within gray matter rostral or caudal to the injury site. Migration of hGDAs in white matter was more extensive than in gray matter, with both types of hGDAs showing extensive migration both rostral and caudal to the injury site, with maximum rostral/caudal migration distances of 3.24 mm/3.96 mm recorded for hGDAs^CNTF^ and 2.52 mm/2.16 mm recorded for hGDAs^BMP^ respectively.

**Figure 3:**
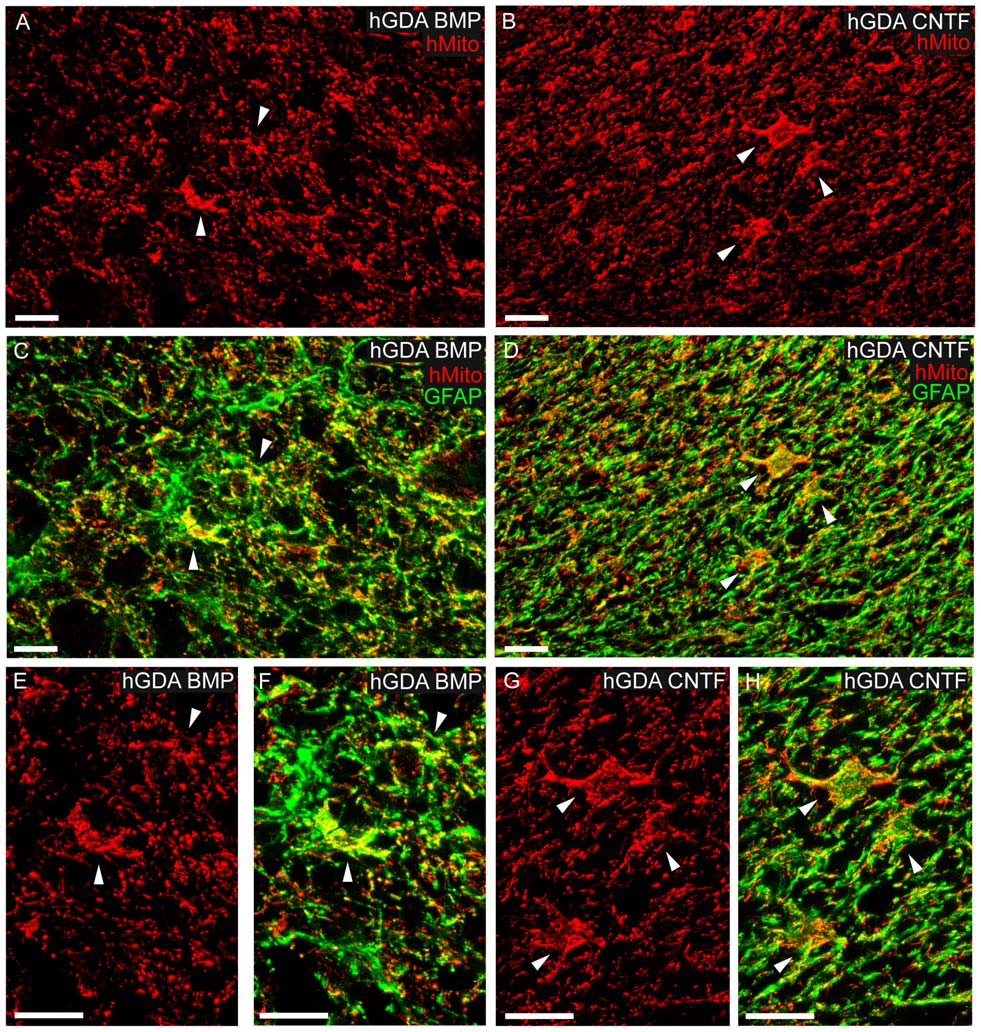
GFAP expression by transplanted hGDAs within DLF transection injuries. Images of hMito+ (red) and GFAP (green) immuno-reactivity within hGDAs^BMP^ (A, C, E, F) and hGDAs^CNTF^ (B, D, G, H) transplants at the center of injury sites showing comparable densities of GFAP+ intermediate filaments (green) within hMito immuno-positive cell bodies and process of both types of hGDAs. Arrowheads indicate some examples of GFAP+/hMito+ hGDA cell bodies at low and high magnification. Images are maximum projections of apotome (Zeiss) optical sections captured through a depth of 3.5 µm of tissue. Survival = 5 weeks post injury/transplantation. All scale bars = 20 µm.

**Figure 4:**
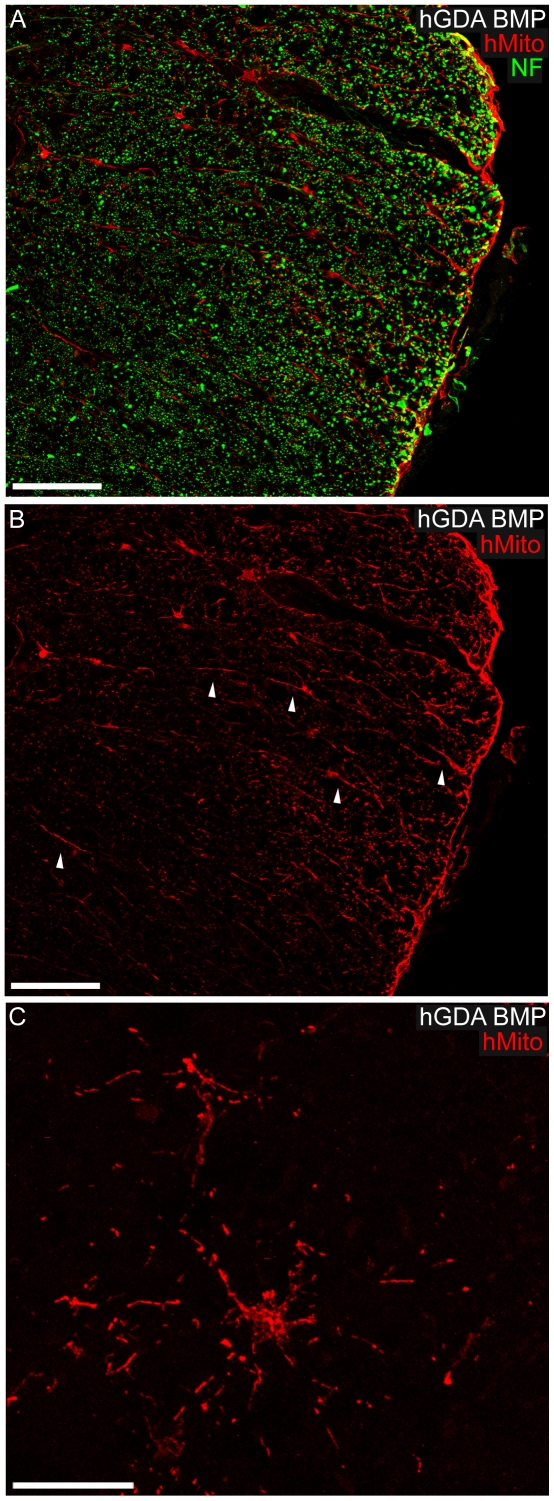
Migration and morphology of hGDAsBMP within DLF white matter. (A, B) High power images showing hMito+ hGDAs^BMP^ in the process of migrating and accumulating at the pial surface within lateral funniculus white matter. Note the elongated radially orientated processes displayed by some hMito+ hGDAs^BMP^ within white matter (B: arrowheads), a glial morphology indicative of tangential migration of these cells towards the adjacent pial surface (hMito: red; NF+ axons: green). (C) hGDAs^BMP^ displaying typical astrocytic “stellate” arrangements of their processes within white matter immediately ventral to the injury site. Survival = 5 weeks post injury/transplantation. Scale bars: A, B = 100 µm; C = 20 µm.

High power imaging of the centers of both types of hGDA grafts showed comparable densities of hMito positive cell bodies and processes that contained GFAP+ intermediate filaments (Fig. 3). Similar densities of hGDAs^BMP^ and hGDAs^CNTF^ had migrated to the pial surface of lateral funiculus white matter both rostral and caudal to the injury site (Figs. 2 C and D; Fig. 4 A and B). The distribution and density of hMito+ mitochondria within individual hGDAs was sufficient to identify their cell bodies and show that both types of hGDA often displayed similar typical astrocytic “stellate” arrangements of their processes within transplant parenchyma, gray matter and white matter (Fig. 2 E and F, Fig. 3 A and B, Fig. 4 C).

### hGDAs^BMP^ promote locomotor recovery while hGDAs^CNTF^ do not

**Figure 5:**
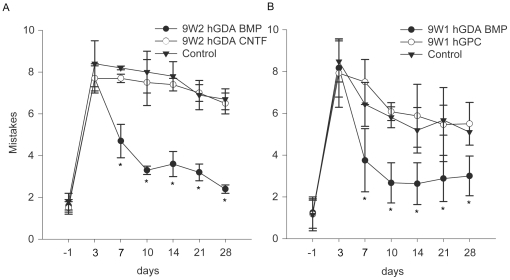
Human GDAs^BMP^ promote robust locomotor recovery but hGDAs^CNTF^ and hGPCs do not. Graphs show the average number of mistakes per experimental group made during Grid walk testing of locomotor recovery at 1 day before injury to 28 days after injury. In two separate experiments, hGDA^BMP^ transplanted animals (closed circles) performed significantly better than hGDA^CNTF^ (A) or hGPC (B) transplanted animals at all time points from 7 to 28 days post injury/transplantation. Note that the performance of hGDA^CNTF^ or hGPC transplanted animals was not significantly different from control injured rats at all time points (two-way repeated measures ANOVA, *p< 0.05).

Despite the similar ability of transplanted hGDAs^BMP^ and hGDAs^CNTF^ to span the rostral to caudal extent of injury sites and migrate into adjacent tissues, only hGDAs^BMP^ promoted locomotor recovery following transplantation into the transected dorsolateral funiculus (DLF). This injury severs descending, supraspinal axons and causes chronic deficits in both fore- and hind-limb motor function [32], which can be detected by the grid-walk behavioral test [33]. A similar average number of mistakes were seen in each group at 3 days post injury/transplantation (Fig. 5 A). At day 7 the hGDA^BMP^ group improved from an average 7.7±0.7 mistakes per crossing to 4.7±0.8, and further improved by day 28 to only 2.4±0.2 mistakes, comparable to uninjured controls. By comparison, by day 28 the untreated injury group and animals receiving hGDAs^CNTF^ transplants made 6.7±0.5 and 6.5±0.5 mistakes respectively. The number of mistakes made by rats in the hGDA^CNTF^ group was not significantly different from those in the media-injected DLF injured control group and did not improve statistically over time. Persistent survival of the transplant was not required in order to obtain benefit after transplantation, as behavioral recovery was as extensive in animals in which transplanted cells were still present at 5 weeks as in those in which no human cells were detected at this time point.

### Pre-differentiation of hGPCs to hGDAs^BMP^ is required for robust functional recovery

We next examined the question of whether the precursor cells from which hGDAs^BMP^ were derived were also capable of promoting behavioral recovery after DLF transection and found that pre-differentiation of these precursor cells into astrocytes was essential to promote significant functional recovery. Rats that received hGDA^BMP^ transplants performed significantly better on the grid-walk test than either the hGPC transplanted group or the media-injected control injury group at all time points from 7 to 28 days post injury/transplantation (Fig. 5 B). The number of mistakes made by the hGPC treated group was not different from the injury control group at all post-injury time points and did not improve statistically over time. Specifically, at 3 days after injury/transplantation the average number of mistakes for each group were 8.2+/−1.3 (media-injected control injury), 7.9+/−1.6 (hGPCs), and 8.2+/−1.4 (9W-1 hGDAs^BMP^), which are not significantly different from each other (as also seen for 9W-2 hGDAs^BMP^). However by day 7 the hGDA^BMP^ group showed a robust improvement to 3.75+/−1.5 mistakes, and by 28 days post injury/transplantation, the last time point tested, the 9W-1 hGDA^BMP^ group made an average of only 3.0+/−0.7 mistakes, compared to 5.5+/−1.0 and 5.1+/−0.8 mistakes for hGPC treated and control groups respectively (p<0.05, Fig. 5 B).

### hGDAs^BMP^ are more effective at providing neuroprotection than hGDAs^CNTF^ and hGPCs

**Figure 6:**
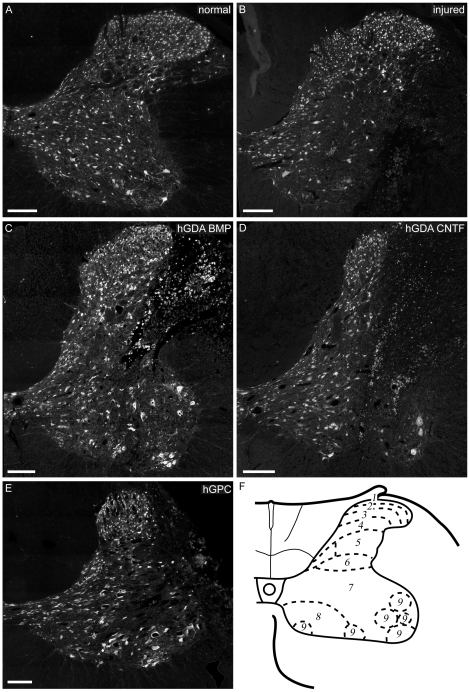
hGDA^BMP^ transplants promote protection of spinal cord neurons while hGDA^CNTF^ and hGPC transplants do not. Montaged images of NeuN immuno-histochemistry at the C5 spinal level of normal (A) and untreated injured (control) spinal cords (B) show that the unilateral DLF transection injury causes loss of NeuN+ neurons in multiple spinal cord laminae adjacent to the transected white matter. Transplantation of hGDAs^BMP^ (C) promotes significant protection of neurons in laminae 7, 8, and 9 at the injury center. In contrast, transplantation of hGDAs^CNTF^ (D) or hGPCs (E) did not promote significant levels of neuroprotection. (F) Schematic showing gray matter laminae at the C5 level of the rat spinal cord (adapted from [39]). All scale bars = 200 µm.

**Figure 7:**
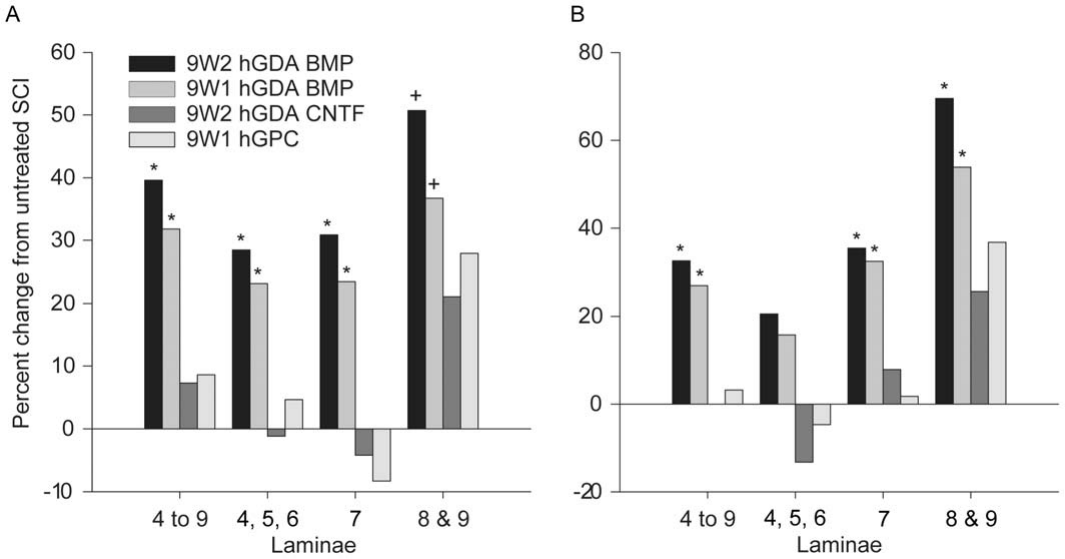
hGDAs^BMP^ promote robust neuroprotection of spinal cord neurons but hGDAs^CNTF^ and hGPCs do not. (A) hGDA^BMP^ transplantation led to significant increases in numbers of NeuN+ neurons counted in a 1.8mm length of spinal cord encompassing the injury site. Graphs show percentage changes in numbers of NeuN+ neurons in laminae 4 to 9; laminae 4, 5, and 6; 7; and 8 and 9 in spinal cords from animals that received transplants of 9W2 or 9W1 hGDAs^BMP^, hGDAs^CNTF^ or hGPCs and untreated control injuries. (B) Analysis of neuron survival within laminae immediately adjacent to the site of injury shows that hGDA^BMP^ transplantation promoted significant protection of neurons when all laminae were considered (4 to 9), with the most robust increases in neuron numbers in intermediate (7) and ventral (8 and 9) gray matter laminae. Numbers of NeuN+ neurons in spinal cords of rats transplanted with GDAs^CNTF^ or hGPCs were not significantly different from each other or untreated spinal cord injuries. * ANOVA and Pairwise Multiple Comparison (Holm-Sidak), p<0.05. + t-test comparison with untreated spinal cord injuries, p<0.05.

Differences in behavioral recovery were mirrored by marked differences in promotion of neuronal survival within ipsilateral gray matter immediately adjacent to sites of injury (Fig. 6 and Fig. 7) and even within rostral and caudal gray matter in which no transplanted hGDAs^BMP^ were observed. Previous studies of neuron survival by the Priestley lab have confirmed with toluidine blue histochemistry that NeuN immuno-reactivity is a reliable marker of neuron survival after spinal cord injury [34]. At 5 weeks post injury/transplantation, NeuN+ neuron cell bodies were counted in laminae 4 to 9 on the injured, right hand side of the spinal cord (Tables 1 and 2). These regions of the spinal cord were chosen because they are either post synaptic targets of the corticospinal (laminae 3–7, dense terminations; laminae 8 and 9, sparse terminations), rubrospinal (laminae 5–7), and raphe (laminae 7–9) supra-spinal motor systems; contain interneurons, propriospinal neurons (laminae 4–8), or commissural neurons (lamina 8) involved in motor control; or primary motor neurons (lamina 9) [35]–[39].

**Table 1:** Survival of NeuN^+^ neurons 5 weeks post injury.

Transplant	Lam. 4 to 9*All sections^#^*	Lam. 4 to 9*Injury site*
No Transplant	2374.25+/−352.84	909.0+/−237.02
9W2 hGDA^BMP^	3313.75+/−238.51*	1204.25+/−138.88*
9W2 hGDA^CNTF^	2548.5+/−175.44	906.0+/−103.94*
9W1 hGDA^BMP^	3128.25+/−327.41*	1153.75+/−127.95*
9W1 hGPC	2578.2+/−71.59	938.6+/−117.48
ANOVA	p<0.001	p<0.05

**Table 2:** Lamina-specific analysis of spinal cord neuron survival.

	Lam. 4, 5, 6		Lam. 7		Lam. 8, 9	
	*All sections*	*Injury site*	*All sections*	*Injury site*	*All sections*	*Injury site*
No Transplant	1191.25+/−259.127	456.50+/−124.66	948.75+/−119.29	330.5+/−74.25	359.25+/−108.53	122.0+/−45.35
9W2 hGDA^BMP^	1530.25+/−139.1*	550.0+/−103.91	1242+/−95.62*	447.5+/−41.99*	541.5+/−61.47†	206.75+/−22.40*
9W2 hGDA^CNTF^	1178.75+/−126.64	396.0+/− 71.58	909.75+/−48.92	356.75+/−12.34	435+/−74.62	153.25+/−42.52
9W1 hGDA^BMP^	1466+/−151.15*	528.50+/−59.04	1171+/−110.22*	437.5+/−49.94*	491.25+/−99.12†	187.75+/−43.87*
9W1 hGPC	1248+/−35.02	435.2+/−61.72	870.4+/−74.65	336.6+/−50.97	459.8+/−70.28	166.8+/−49.43
ANOVA	p<0.05	p = 0.09	p<0.001	p<0.05	p = 0.08	p<0.05

# All sections sampled above, at and below injury site.

* Significantly different from SCI animals receiving no transplant, Holm-Sidak post hoc test.

† Separate t-tests demonstrate that 9W1 and 9W2 GDAs^BMP^ are statistically different from control injury (p<0.05).

Significant improvements in neuronal survival in all laminae studied were seen in a 1.8 mm length of spinal cord encompassing the injury site of hGDA^BMP^-treated animals. Combined neuron counts for all laminae (4 to 9) showed that hGDA^BMP^ transplantation promoted increases of 40% and 32%, in two separate experiments, of surviving neurons compared to untreated injured spinal cords (Fig. 7 A; Table 1), with significant increases seen in all laminae. Analysis of a region closer to the site of injury, through 750 µm of tissue spanning the injury center, revealed notably robust increases in numbers of neurons for lamina 7 (35% and 32%) and laminae 8 and 9 (70% and 54%) above control injured cords (Fig. 7 B; Table 2). As with behavioral recovery, continued graft survival was not required for promotion of neuronal survival. Significant increases in neuron numbers were not observed in lamina 4, 5, 6 adjacent to injury centers, despite rescue of neurons more distal to the zone of injury; an outcome most likely due to neuron loss resulting from direct trauma to these laminae at time of injury.

## Discussion

The present studies provide multiple novel findings relevant to the development of astrocyte transplantation therapies for treatment of the injured or diseased central nervous system. We show that subpopulations of human astrocytes, generated by activation of different signaling pathways in the same population of human glial precursor cells, have markedly different effects when transplanted into the injured spinal cord. hGDAs^BMP^ provided extensive benefit, including robust protection of spinal cord neurons, increased support of axon growth and locomotor recovery. In contrast, transplantation of either undifferentiated hGPCs or hGDAs^CNTF^ failed to provide significant benefits. The major gains in behavioral recovery and neuronal survival achieved by *pre*-differentiation of glial precursors to specific, beneficial astrocytic cell types prior to transplantation stresses the need to consider such manipulations as a critical component in the optimization of stem/precursor cell transplantation based therapies.

The development of astrocyte transplantation represents a new avenue for the treatment of CNS injury, as contrasted with the extensive research that has been conducted on replacement of oligodendrocytes. Starting with transplants of human oligodendrocytes in the late 1980s [40], and more recently with populations of human oligodendrocyte progenitor cells isolated from the developing or adult CNS, or from human embryonic stem cells, it has been possible to generate extensive myelination upon transplantation into spinal cord injury or into congenital mouse models of hypomyelination [41]–[48]. In contrast, much less is known about the potential utility of astrocyte-based therapies. Moreover, initial studies showed only modest benefits of astrocyte transplantation for treatment of traumatic injury to the spinal cord [49]–[53].

One of the striking differences in outcome between our studies and work on oligodendrocyte and oligodendrocyte-precursor replacement lies in the finding that differentiation of precursor cells into a specific astrocyte subtype *prior* to transplantation provides a much greater level of benefit than transplantation of the precursor cells themselves. This is the opposite situation to that reported in the oligodendrocyte lineage, for which a greater degree of pre-transplant differentiation is associated with less effective repair [54], [55]. While it may be that precursor cell transplantation is of potential use in astrocyte replacement in neurological disorders such as ALS [56], our results demonstrate the importance of determining whether direct transplantation of astrocytes themselves provides greater benefit. In light of the modest benefits obtained with transplantation of rodent astrocytes isolated directly from the immature CNS [49]–[53], however, our present and earlier studies [14], [57] suggest that it is necessary instead to transplant astrocytes generated from precursor cells in vitro in order to optimize benefit.

Along with demonstrating the marked benefits from astrocyte transplantation in experimental injuries of the spinal cord, our studies also demonstrate that obtaining benefit may require transplanting very specific populations of human astrocytes. The significant difference in outcome achieved by transplantation of hGDAs^BMP^ versus hGDAs^CNTF^ demonstrates clearly that not all astrocytes are equivalent in respect to their therapeutic value, and this appears to be the first study demonstrating functional differences between different human astrocyte populations with respect to repairing the adult central nervous system. It is also interesting to note the similarity between the outcomes obtained with human cells and with our prior studies on rat cells [14], [57]. In a similar fashion to that observed for rodent GDAs transplanted hGDAs^BMP^ were more supportive of axon growth than hGDAs^CNTF^ at sites of spinal cord injury. Like the human GDAs^BMP^, rodent derived GDAs^BMP^ promote robust functional recovery, while GDAs^CNTF^ did not [57]. The conservation of the phenotypic and functional properties of GDAs^BMP^ and GDAs^CNTF^ between human astrocytes and rat astrocytes suggests that such properties are fundamental to the biology of these cells. The one difference observed in these studies was that hGDA^BMP^ transplantation showed a slightly longer delay (7 days versus 3 days) in providing significant behavioral recovery. Whether this is due to differences in cell properties or a consequence of the xenograft itself remains to be investigated.

It was also of interest to observe that prolonged survival of the grafted astrocytes was not required to obtain durable improvements in behavior and neuronal survival. This also demonstrates a conservation of outcomes between human cells and rat cells, which also did not require prolonged survival to provide durable benefit [14], [57], suggesting that this too might be a conserved aspect of GDA^BMP^ function.

This is also the first study, to our knowledge, in which transplanted astrocytes (rodent or human) have been shown to promote extensive neuroprotection of spinal cord neurons following spinal cord injury, an observation consistent with the robust neuroprotective effects of intra-spinal rodent GDA^BMP^ transplants on axotomized neurons of the red nucleus [14], [57]. While future studies will reveal whether transplantation of hGDAs^BMP^ to DLF injuries provide protection of red nucleus neurons, hGDAs^BMP^ provided robust neuron protection in multiple spinal cord laminae, even in more distant gray matter in which there was no evidence of hGDAs^BMP^ migration. hGDAs^BMP^ were able to promote survival of multiple neuronal populations within multiple gray matter laminae with notably robust increases of up to 69% in neuronal survival in laminae 8 and 9 containing motor neurons. In contrast, although SCI rats that were treated with hGPCs and hGDAs^CNTF^ showed positive trends in neuron protection for laminae 8 and 9, this did not translate to improvements in grid-walk performance and these cells failed to promote statistically significant increases in neuronal survival even in laminae into which they had migrated.

The underlying mechanisms accounting for why hGDAs^BMP^ are so much more beneficial in terms of neuroprotection and functional recovery than either hGDAs^CNTF^ or undifferentiated precursor cells when transplanted into spinal cord injured rats remain to be investigated, but it is likely that multiple cellular functions are involved. For example, hGDAs^BMP^ express higher levels of such astrocyte-related genes as glutamate transporter 1, connexin 43, and AKAP12, which are relevant to maintaining tissue homeostasis in the CNS as well as reducing astrogliosis and neuronal death after injury, mediating glutamate uptake and promoting blood-brain barrier formation [23], [24], [58]–[61]. These and other differences between hGDAs^BMP^ and undifferentiated hGPCs and hGDAs^CNTF^, such as the marked differences in expression of axon growth inhibitory CSPGs and the expression of GDNF, may all contribute to creating a particularly effective cell type for promoting functional recovery in the traumatically injured adult central nervous system.

In brief our present studies provide the first demonstration of the utility of human astrocyte transplantation as a therapy for central nervous system injuries. Moreover, our studies provide a specific population of human astrocytes that appear to be particularly suitable for further development towards clinical applications.

## Materials and Methods

### Ethics Statement

The University of Rochester RSRB has reviewed this study and determined that based on federal (45 CFR 46.102) and University criteria, the study does not qualify as human subjects research and has waived the need for consent (RSRB#00024759). All animal procedures were performed under guidelines of the National Institutes of Health and approved by the Institutional Animal Care and Utilization Committee (IACUC) of University of Colorado Denver, Aurora, CO (UCAR# 80710(05)1E). or the IACUC of University of Rochester Medical Center, Rochester, NY (UCAR# 2008-075).

### Preparation of human cells

Human spinal cord tissues were obtained from two nine week old, de-identified abortus samples collected in the course of medically prescribed procedures using the Safe-Harbor Method. Spinal cord-derived glial precursors were grown and isolated as previously described [62]–[64]. Spinal cord tissue was dissected from the rostral neural tube of two 9 week old samples (referred to here as 9W1 and 9W2). After removal of the meninges, tissue was digested at 37°C with 59 U/ml papain (Worthington) in Hanks balanced salt solution (HBSS, Invitrogen) supplemented with 10 mM Hepes (EMD), pH 8.0 and 125 U/ml Dnase I (Sigma), and triturated in 0.3%(w/v) BSA/HBSS (Sigma), 250 U/ml Dnase I. A2B5^+^PSA-NCAM**^−^** glial progenitor cells were isolated by step-wise immunopurification using anti-PSA-NCAM and A2B5-bound magnetic beads (Miltenyi). and cultured in 5% O_2_/7% CO_2_ in Bottenstein-Sato F12 medium with 10 ng/ml human recombinant basic fibroblast growth factor (Peprotech) on a substrate of 1 µg/cm^2^ fibronectin (Chemicon) and 0.5 µg/cm^2^ laminin (Invitrogen). Differentiation of hGPCs was induced at 2500 cells/cm^2^ by replacing bFGF with either 20 ng/ml BMP-4 (R&D) or 10 ng/ml CNTF (Peprotech) and allowed to differentiate for 7 days prior to harvest.

### RNA and protein expression analysis

Characterization of hGPC and hGDA cultures was performed by reverse-transcriptase semi-quantitative polymerase chain reaction (RT-QPCR), Western blot and immunofluorescent labeling as previously described [7], [65]. Multiplex QPCR reactions of RT product were performed using FAM-labeled probes for aquaporin 4, S100b, CX43, GLT-1 and AKAP12, in combination with VIC-labeled, primer limited GAPDH probe and Taqman mastermix (all Applied Biosystems). DDCt analysis was performed using Microsoft Excel software as previously described [66]. Independent experiments were performed in triplicate and average fold change expression normalized to expression in undifferentiated hGPCs grown in bFGF. Western blot analysis of chondroitin sulfate proteoglycans and Olig2 was performed as previously described [57], using anti-phosphacan monoclonal (1∶1000, 3F8, Developmental Studies Hybridoma Bank), anti-CSPG4/NG2 monoclonal (1∶2000, Chemicon), anti-Olig2 polyclonal (1∶4000, Chemicon) and anti-b-tubulin monoclonal antibody (1∶1000, Santa Cruz). Expression levels of phosphacan (320–340 kDa band) NG2 (270–300 kDa band) and Olig2 (32 kDa band), respectively, were normalized for each sample to β-tubulin (52 kDa) expression. All Western blot experiments were conducted in triplicate and results were compared using the Student's t-test, p<0.05. Immunofluorescent labeling was performed using anti-Olig2 (1∶4000, Chemicon) and anti-GFAP (1∶400, Cell Signaling) followed by fluorescently labeled, secondary anti-Ig antibodies (Alexa 488 and 568 conjugates, Invitrogen) at a 1∶2000 dilution. Monochrome images of parallel samples were captured using identical exposure times and gain settings, and merged as pseudo-colored images.

### Spinal cord injury model

Adult female Sprague Dawley rats (3 months old, Harlan) were used in all *in vivo* spinal cord injury experiments and were anesthetized by injection of a cocktail containing ketamine and xylazine. Unilateral transections of the right-side dorso-lateral funiculus (DLF) including the rubrospinal pathway were conducted at the C3/C4 intervertebral spinal cord level (Supplemental Fig. S1A and S1B). The dorsal surface of the spinal cord was exposed by opening the intervertebral space between the C3 and C4 vertebrae. After opening the dura, a 1 mm deep transection was made lateral to the midline using micro-scissors. To ensure that the injury was complete and that the depth was uniformly 1 mm, a 30 gauge needle was again inserted into the transection site to a depth of 1 mm and slowly passed through the medial to lateral extent of the injury. The use of an inter-vertebral surgery approach in combination with discreet transection injuries of the dorsolateral funiculus results in highly consistent deficits in grid-walk locomotor performance [14], [57].

A total of 6 µl of hGDA^BMP^, hGDA^CNTF^ or hGPC suspensions (30,000 cells/µl; 180,000 cells total) per animal were acutely transplanted into six different sites at the injury site on the right side of the spinal cord: medial and lateral of the rostral and caudal injury margins, and medial and lateral of the injury center (Supplemental Fig. S1C). Control injured rats were injected with 6 µl HBSS. All control or cell transplanted rats were immune suppressed. Rats in the 9W2 groups were given daily injections of cyclosporine (1 mg/100 g body weight) beginning the day before injury/transplantation through the duration of the experiment. Due to a temporary unavailability of injectable cyclosporine, rats in the 9W1 group were given a bolus injection of methyl prednisolone (30 mg/kg body weight) 1 hour prior to injury/transplantation.

### Histology

At 5 weeks post-surgery animals were deeply anesthetized and transcardially perfused with 0.1 M PBS followed by 4% paraformaldehyde in 0.1M PBS. Dissected spinal cords were cryosectioned and immunofluorescently labeled as previously described [14], [57]. The following primary antibodies were used: mouse anti-GFAP (Sigma), rabbit polyclonal anti-GFAP (Sigma) or goat anti GFAP (Lifespan Biosciences); mouse anti-NeuN (Millipore); rabbit anti- 200 kD neurofilament (Serotec); mouse anti-human mitochondrial antigen (Millipore). Alexa-488, Alexa-594, and Alexa-647 conjugated secondary antibodies (Invitrogen) were used to visualize primary antibody binding. All secondary antibodies were pre-absorbed against rat serum. To control for nonspecific secondary antibody binding, adjacent sections were also processed as described above without primary antibodies. Some sections were counterstained with DAPI to show nuclei. Labeled sections were examined and imaged using a Zeiss 510 Meta confocal microscope. Antigen co-localization and cellular associations were determined with Zeiss Confocal image analysis software.

### Quantification of axon growth into GDA treated injury sites

The relative density of axons within the centers of hGDA^BMP^ or hGDA^CNTF^ transplanted injury sites was determined by quantifying neurofilament-immunoreactive pixels in 4 tissue sections per spinal cord from 5 animals per experimental group. Images were captured (Zeiss-Z1 microscope) of the right-side dorsolateral funiculus from every sixth histological cross section (4 sections in total per spinal cord) from tissue at injury centers. Using Image-J analysis software, a 465 µm×465 µm square region of interest was drawn on each image with the upper right corner located on the dorso-lateral outer edge of the transplant mass such that the region of interest was contained within injury sites/transplant parenchyma (Supplemental Fig. S2). The area within the region of interest filled with NF+ pixels was determined. The sum total of NF+ areas of each region of interest per spinal cord was calculated, and then the average area per experimental group was determined. A two-sample T-test was applied to assess statistical significance (alpha = 0.05).

### Quantification of neuron survival

Neuronal survival within spinal cord gray matter was determined using NeuN immuno-reactivity as a marker of surviving spinal cord neurons after spinal cord injury [34]. To quantify surviving NeuN+ neurons, 15 sections per spinal cord at 5 weeks post injury were sampled from a 1.8 mm length of spinal cord encompassing the injury site. 5 spinal cords per experimental group were analyzed. Starting 400 µm rostral to the injury site, the right side of every sixth serial section was imaged (Zeiss-Z1 microscope). Using AxioVision software the gray matter was subdivided into 3 regions: laminae 4-6; lamina 7; laminae 8–9. NeuN+ neurons were counted within each region per section. The average number of neurons (+/− one standard deviaton) per total number of neurons was determined per region (tables 1 and 2). The percent change was calculated by dividing the average number of neurons per group by the average number of neurons in the injury control group. A second set of calculations was conducted on 6 sections per spinal cord through 750 µm of tissue spanning the injury center. One way ANOVA and Holm-Sidak multiple comparisons post hoc tests were applied to determine statistical significance (p<0.05). The power of these tests, with an alpha of 0.05, was 0.996 when all sections are considered, and 0.702 when only the 6 sections spanning the injury center are considered.

### Analysis of Locomotor Recovery

Behavioral analysis of volitional foot placement was tested using a grid-walk behavioral test (Foot Misplacement Apparatus, Columbus Instruments) as previously described [14], [57]. Two weeks before surgery, rats were trained to walk across a horizontal ladder and those that consistently crossed without stopping were used in experiments. Baseline gridwalk scores were obtained one day prior to surgery and rats were randomly assigned into experimental groups. One experiment included the following three groups: DLF injury + media injection (n = 8), DLF injury + 9W-1 hGPCs (n = 8), and DLF injury + 9W-1 hGDAs^BMP^ (n = 8). The second experiment included DLF injury + media injection (n = 6), DLF injury + 9W-2 hGDAs^CNTF^ (n = 6) and DLF injury + 9W-2 hGDAs^BMP^ (n = 7). At 3, 7, 10, 14, 21, and 28 days post-surgery, each rat was tested three times and the number of mistakes from each trial was averaged to generate a daily score for each animal. Two-way repeated measures ANOVA and Holm-Sidak post hoc (p<0.05) were applied to assess statistical significance using Sigma Stat 3.5 (Systat Software Inc.). The power of these tests for each independent behavior study, with an alpha of 0.05, was equal to 1.0.

## Supporting Information

Figure S1 Schematic illustrations of the adult rat dorso-lateral funiculus (DLF) transection spinal cord injury model and cell injections at injury sites. Dorsal (A) and cross section (B) schematics of the rat cervical spinal cord showing right side unilateral transection injury (red shaded area) conducted at the level of the C3/C4 intervertebral junction. (C) Injections (six in total) of either hGDAs or hGPCs were made at sites if injury, two into injury centers and two further injections each to rostral and caudal injury margins respectively (black diamonds represent injection sites). C3/C4, junction of the third and fourth cervical vertebrae; DLF, dorsolateral funiculus; Cf, cuneate fasciculus; Gf, gracile fasciculus; GM, gray matter. (Cross section schematic (B) adapted from Grant and Koerber [39]).
(TIF)

Figure S2 Schematic illustration of neurofilament sampling region at injury/transplantation sites. Image-J analysis software has been used to draw a 465 µm×465 µm square region of interest (ROI, white box) on a representative image of an NF immuno-stained tissue section at the center of hGDA^CNTF^ treated DLF injury site. The upper right corner of the ROI is located on the dorso-lateral outer edge of the transplant mass such that that the region of interest is contained within the injury site/transplant mass. Scale bar = 200 µm.
(TIF)
