# Supplementary figures and images for: Transplantation of Specific Human Astrocytes Promotes Functional Recovery after Spinal Cord Injury
Source: PLoS One. 2011 Mar 2;6(3):e17328. doi: 10.1371/journal.pone.0017328 (PMC3047562; doi:10.1371/journal.pone.0017328)

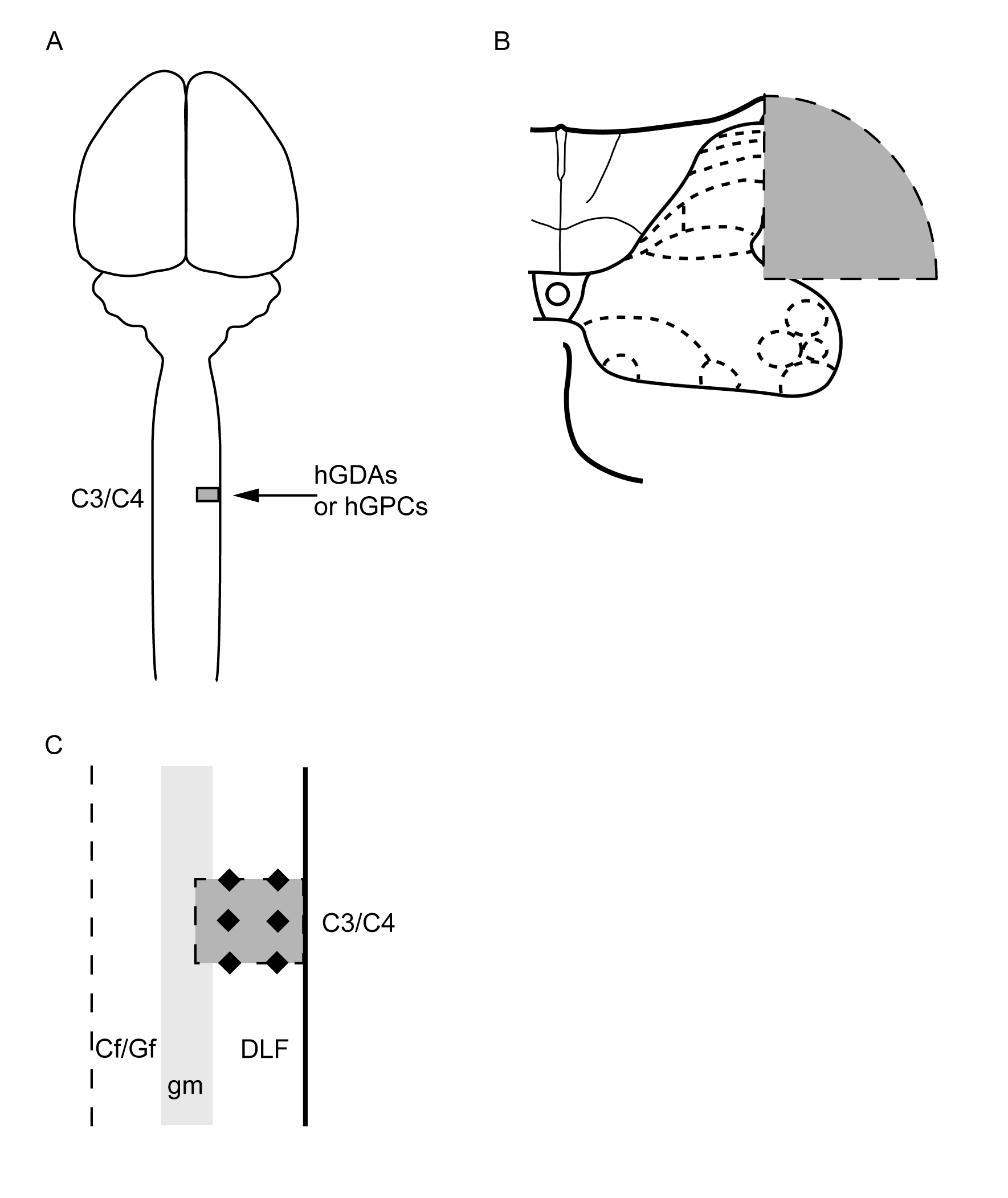

Supplement: Figure S1 — Schematic illustrations of the adult rat dorso-lateral funiculus (DLF) transection spinal cord injury model and cell injections at injury sites. Dorsal (A) and cross section (B) schematics of the rat cervical spinal cord showing right side unilateral transection injury (red shaded area) conducted at the level of the C3/C4 intervertebral junction. (C) Injections (six in total) of either hGDAs or hGPCs were made at sites if injury, two into injury centers and two further injections each to rostral and caudal injury margins respectively (black diamonds represent injection sites). C3/C4, junction of the third and fourth cervical vertebrae; DLF, dorsolateral funiculus; Cf, cuneate fasciculus; Gf, gracile fasciculus; GM, gray matter. (Cross section schematic (B) adapted from Grant and Koerber [39]). (TIF) [file pone.0017328.s001.tif]

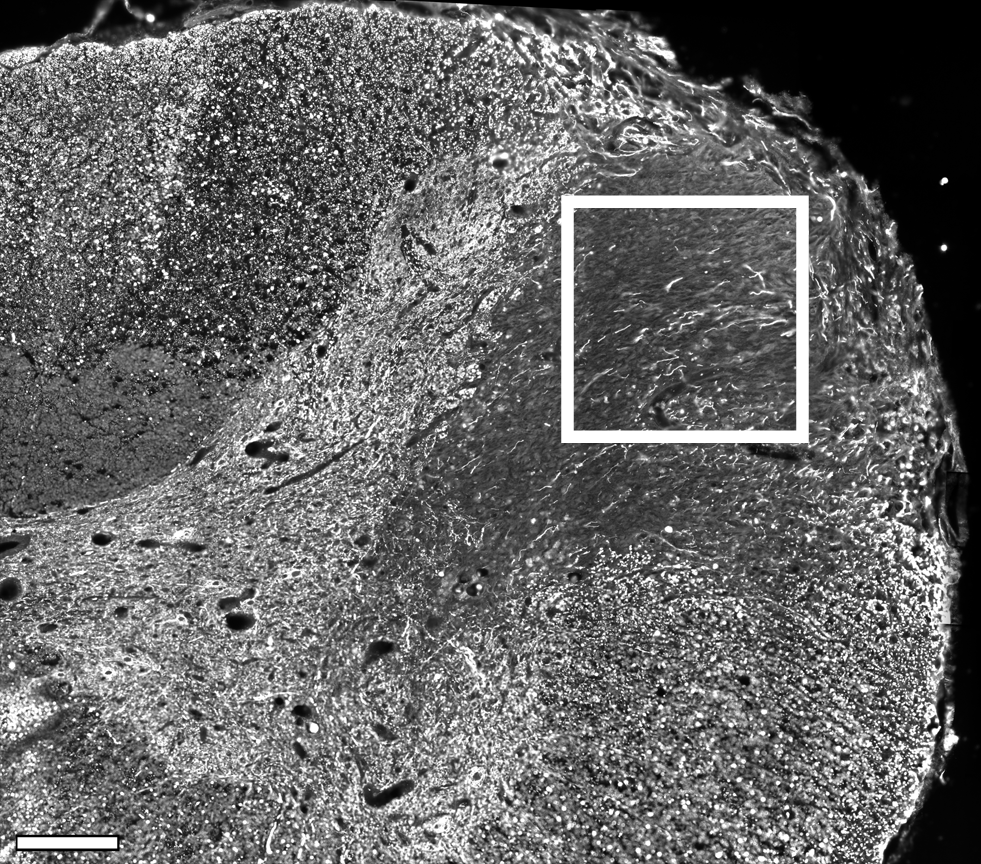

Supplement: Figure S2 — Schematic illustration of neurofilament sampling region at injury/transplantation sites. Image-J analysis software has been used to draw a 465 µm×465 µm square region of interest (ROI, white box) on a representative image of an NF immuno-stained tissue section at the center of hGDACNTF treated DLF injury site. The upper right corner of the ROI is located on the dorso-lateral outer edge of the transplant mass such that that the region of interest is contained within the injury site/transplant mass. Scale bar = 200 µm. (TIF) [file pone.0017328.s002.tif]
